# Cystic Fibrosis‐Related Diabetes and Celiac Disease in a Paediatric CF Patient: Presence of CFRD‐Associated SNPs rs7903146 (TT) and rs4077468 (GG)

**DOI:** 10.1002/rcr2.70551

**Published:** 2026-03-16

**Authors:** Eka Kvaratskhelia, Dodo Agladze, Nino Vardosanidze, Mariam Ghughunishvili, Sandro Surmava, Elene Abzianidze, Tinatin Tkemaladze

**Affiliations:** ^1^ Department of Molecular and Medical Genetics Tbilisi State Medical University Tbilisi Georgia; ^2^ V. Bakhutashvili Institute of Medical Biotechnology Tbilisi State Medical University Tbilisi Georgia; ^3^ Petre Shotadze Tbilisi Medical Academy Tbilisi Georgia; ^4^ Medical Genetics and Laboratory Diagnostics Center Tbilisi Georgia; ^5^ G. Zhvania Pediatric University Clinic Tbilisi State Medical University Tbilisi Georgia; ^6^ Ivane Beritashvili Center of Experimental Biomedicine Tbilisi Georgia

**Keywords:** celiac disease, CFRD, cystic fibrosis, rs4077468, rs7903146

## Abstract

Cystic fibrosis‐related diabetes (CFRD) is a common metabolic complication in adolescents with cystic fibrosis (CF). Variants in *TCF7L2* and *SLC26A9* have been implicated in CFRD susceptibility. The coexistence of CF, CFRD, and celiac disease in childhood is rare and presents diagnostic and therapeutic challenges. We describe a 14‐year‐old female with CF carrying compound heterozygous *CFTR* mutations (p.Glu831Ter and *p.Ser573Phe*). Sweat chloride values were borderline. At age 7, she was diagnosed with celiac disease after elevated anti‐tTG IgA (297 U/mL) and IgG (22.6 U/mL) and was started on a gluten‐free diet. At age 12 years, she presented with diabetic ketoacidosis and was diagnosed with CFRD, requiring insulin therapy. Genotyping revealed high‐risk diabetes and CF‐modifier variants: rs7903146 TT (*TCF7L2*) and rs4077468 GG (*SLC26A9*). This case highlights the interplay of CF, autoimmunity, and genetic predisposition in early onset CFRD. Genetic profiling may improve risk assessment in CF patients with complex endocrine or autoimmune presentations.

## Graphical abstract

This case describes a 14‐year‐old girl with cystic fibrosis who developed both celiac disease and early‐onset cystic fibrosis–related diabetes (CFRD). Genotyping revealed high‐risk variants in TCF7L2 (rs7903146 TT) and SLC26A9 (rs4077468 GG), suggesting a strong genetic contribution to her metabolic deterioration. Despite the coexistence of CF, autoimmunity, and endocrine dysfunction, timely diagnosis and management led to preserved growth and stable lung function.

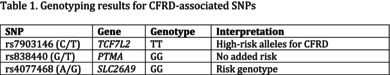

## 1 Introduction

Cystic fibrosis (CF) is a multisystem autosomal recessive disorder caused by pathogenic variants in the *CFTR* gene. Endocrine complications, particularly cystic fibrosis‐related diabetes (CFRD), develop in 19% of adolescents and up to 50% of adults with CF. CFRD arises from β‐cell dysfunction due to pancreatic scarring, chronic inflammation, malnutrition, and genetic susceptibility [1].

Polymorphisms in *TCF7L2*, particularly rs7903146, are among the strongest determinants of diabetes risk. Variants in *SLC26A9*, a CF‐modifier gene involved in chloride transport, also contribute to CFRD susceptibility. *PTMA* variants (rs838455, rs838440) may affect insulin sensitivity and are associated with CFRD [2, 3]. Celiac disease is more common in CF than in the general population and can exacerbate malabsorption and metabolic instability [4].

The combination of CF, CFRD, and celiac disease in childhood is unusual. This report describes such a triad and emphasises the potential contribution of *TCF7L2* and *SLC26A9* variants to early CFRD onset.

## 2 Case Report

The patient is a 14‐year‐old female with a history of cystic fibrosis (CF), celiac disease, and cystic fibrosis‐related diabetes (CFRD). She was born to non‐consanguineous parents. She first presented at age 3 years with chronic cough, steatorrhea, and poor weight gain in the Medical Genetics and Laboratory Diagnostic Center, Tbilisi, Georgia. Genetic analysis identified compound heterozygosity for *CFTR* mutations p.Glu831Ter and *p.Ser573Phe* (c.2491G>T/c.1718C>T), confirming CF. Sweat chloride values were borderline (36.4 and 31.0 mmol/L), yet her clinical phenotype was consistent with CF. She was started on pancreatic enzyme replacement and inhaled hypertonic saline ≥ 3%. Serial microbiology remained negative.

At age 7 years, she developed gastrointestinal symptoms and was diagnosed with celiac disease after markedly elevated anti‐tissue transglutaminase IgA (297 U/mL) and IgG (22.6 U/mL). A strict gluten‐free diet led to clinical improvement and stabilisation of nutritional status.

At 12 years, she presented with fatigue, polyuria, and vomiting. Laboratory evaluation confirmed diabetic ketoacidosis, requiring hospitalisation, intravenous fluids, and insulin therapy. She fulfilled diagnostic criteria for CFRD, and after stabilisation was transitioned to daily subcutaneous insulin. Review of her earlier glucose values showed fluctuating glycemia (110 mg/dL in 2021 and 64 mg/dL in 2023), indicating a progressive decline in glucose tolerance prior to the event.

To evaluate genetic contributions to early CFRD, three diabetes‐related SNPs were analysed using the TaqMan SNP genotyping method (Thermo Scientific, USA). She carried rs7903146 TT (*TCF7L2*), the highest‐risk genotype for impaired insulin secretion; rs4077468 GG (*SLC26A9*), a CF‐modifier gene associated with increased CFRD risk; and rs838440 GG (*PTMA*), a non‐risk genotype (Table 1).

**TABLE 1:** Genotyping results for CFRD‐associated SNPs.

SNP	Gene	Genotype	Interpretation
rs7903146 (C/T)	*TCF7L2*	TT	High‐risk alleles for CFRD
rs838440 (G/T)	*PTMA*	GG	No added risk
rs4077468 (A/G)	*SLC26A9*	GG	Risk genotype

At her most recent follow‐up (age 14 years 2 months), she demonstrated favourable growth with height 174 cm (*z*‐score 2.03), weight 63 kg (*z*‐score 1.1), and BMI 20.81 (*z*‐score 0.43). Spirometry showed FEV_1_; and FVC around −1.0 *z*‐score. She remained stable on pancreatic enzymes, gluten‐free diet, and daily insulin therapy. (Table 1).

## 3 Discussion

CFRD is a distinct form of diabetes characterised primarily by insulin insufficiency [1]. Although pancreatic fibrosis plays a central role, genetic modifiers influence disease onset and severity. The *TCF7L2* rs7903146 TT genotype is strongly associated with impaired insulin secretion, decreased incretin response, and increased diabetes susceptibility. The patient's rs4077468 GG genotype in *SLC26A9* adds further CFRD risk through altered epithelial chloride transport and CF‐related β‐cell stress [2, 3].

The combination of these risk alleles likely contributed to the patient's early CFRD onset and presentation with ketoacidosis, a relatively uncommon but recognised manifestation in adolescents with CF [5].

Celiac disease, diagnosed at age 7, introduced additional metabolic stress through malabsorption and chronic inflammation [4]. Nevertheless, early diagnosis, adherence to a gluten‐free diet, and comprehensive CF care resulted in preserved lung function and normal growth parameters.

This case emphasises that genetic modifiers should be considered when evaluating early metabolic deterioration in CF patients. Genotype‐informed risk stratification may improve prevention, monitoring, and therapeutic strategies for CFRD.

This paediatric case underscores the complexity created by the coexistence of CF, CFRD, and celiac disease. The presence of high‐risk rs7903146 TT and rs4077468 GG likely contributed to early CFRD onset. Genetic profiling may improve risk stratification and anticipatory guidance for CF patients with multifactorial endocrine complications.

## Author Contributions

Conceptualisation: E.K. and T.T. Data collection: D.A. and N.V. Genetic analysis: S.S. and M.G. Manuscript drafting: E.K. and E.A. All authors reviewed and approved the final manuscript.

## Funding

This work was supported by Shota Rustaveli National Science Foundation (22‐2601).

## Consent

The authors declare that written informed consent was obtained for the publication of this manuscript and accompanying images using the consent form provided by the Journal.

## Conflicts of Interest

The authors declare no conflicts of interest.

## Data Availability

The data that support the findings of this study are available from the corresponding author upon reasonable request.

## References

[1] S. M. Blackman , C. W. Commander , C. Watson , et al., “Genetic Modifiers of Cystic Fibrosis‐Related Diabetes,” Diabetes 62, no. 10 (2013): 3627–3635, 10.2337/db13-0510. 23670970 PMC3781476

[2] S. Hasan , S. Soltman , C. Wood , and S. M. Blackman , “The Role of Genetic Modifiers, Inflammation and CFTR in the Pathogenesis of Cystic Fibrosis‐Related Diabetes,” Journal of Clinical & Translational Endocrinology 27 (2021): 100287, 10.1016/j.jcte.2021.100287. 34976741 PMC8688704

[3] M. A. Aksit , R. G. Pace , B. Vecchio‐Pagán , et al., “Genetic Modifiers of Cystic Fibrosis‐Related Diabetes Have Extensive Overlap With Type 2 Diabetes and Related Traits,” Journal of Clinical Endocrinology and Metabolism 105, no. 5 (2020): 1401–1415, 10.1210/clinem/dgz102. 31697830 PMC7236628

[4] J. Walkowiak , A. Blask‐Osipa , A. Lisowska , et al., “Cystic Fibrosis Is a Risk Factor for Celiac Disease,” Acta Biochimica Polonica 57, no. 1 (2010): 115–118. 20300660

[5] S. M. O'Riordan , P. D. Robinson , K. C. Donaghue , and A. Moran , “Management of Cystic Fibrosis‐Related Diabetes in Children and Adolescents,” Pediatric Diabetes 10, no. 12 (2009): 43–50, 10.1111/j.1399-5448.2009.00587.x. 19754617

